# The Effect of Growth Parameters on Electrophysical and Memristive Properties of Vanadium Oxide Thin Films

**DOI:** 10.3390/molecules26010118

**Published:** 2020-12-29

**Authors:** Roman V. Tominov, Zakhar E. Vakulov, Vadim I. Avilov, Daniil A. Khakhulin, Nikita V. Polupanov, Vladimir A. Smirnov, Oleg A. Ageev

**Affiliations:** 1 Institute of Nanotechnologies, Electronics and Electronic Equipment Engineering, Southern Federal University, 347922 Taganrog, Russia; tominov@sfedu.ru (R.V.T.); avilovvi@sfedu.ru (V.I.A.); dhahulin@sfedu.ru (D.A.K.); npolupanov@sfedu.ru (N.V.P.); ageev@sfedu.ru (O.A.A.); 2 Research and Education Center “Nanotechnologies” at the Southern Federal University, Southern Federal University, 347922 Taganrog, Russia; 3 Federal Research Centre The Southern Scientific Centre of the Russian Academy of Sciences, 344006 Rostov-on-Don, Russia; vakulov@ssc-ras.ru

**Keywords:** memristor, ReRAM, resistive switching, pulsed laser deposition, vanadium oxide thin films, neuromorphic systems

## Abstract

We have experimentally studied the influence of pulsed laser deposition parameters on the morphological and electrophysical parameters of vanadium oxide films. It is shown that an increase in the number of laser pulses from 10,000 to 60,000 and an oxygen pressure from 3 × 10^−4^ Torr to 3 × 10^−2^ Torr makes it possible to form vanadium oxide films with a thickness from 22.3 ± 4.4 nm to 131.7 ± 14.4 nm, a surface roughness from 7.8 ± 1.1 nm to 37.1 ± 11.2 nm, electron concentration from (0.32 ± 0.07) × 10^17^ cm^−3^ to (42.64 ± 4.46) × 10^17^ cm^−3^, electron mobility from 0.25 ± 0.03 cm^2^/(V·s) to 7.12 ± 1.32 cm^2^/(V·s), and resistivity from 6.32 ± 2.21 Ω·cm to 723.74 ± 89.21 Ω·cm. The regimes at which vanadium oxide films with a thickness of 22.3 ± 4.4 nm, a roughness of 7.8 ± 1.1 nm, and a resistivity of 6.32 ± 2.21 Ω·cm are obtained for their potential use in the fabrication of ReRAM neuromorphic systems. It is shown that a 22.3 ± 4.4 nm thick vanadium oxide film has the bipolar effect of resistive switching. The resistance in the high state was (89.42 ± 32.37) × 10^6^ Ω, the resistance in the low state was equal to (6.34 ± 2.34) × 10^3^ Ω, and the ratio R_HRS_/R_LRS_ was about 14,104. The results can be used in the manufacture of a new generation of micro- and nanoelectronics elements to create ReRAM of neuromorphic systems based on vanadium oxide thin films.

## 1. Introduction

The biological brain has several advantages over traditional computing systems, the most important of which are learning, generalization, abstraction, and applicability [1,2,3]. Most computers are based on von Neumann architecture [4,5]. However, the architecture faces a limitation called “von Neumann bottleneck”—physical limitation of the information transfer between the central processor and memory block [6,7]. Finally, this led to a slowdown in the development of computing systems in terms of speed and power consumption. Therefore, the attention of scientists was directed to the creation of computing systems with a fundamentally different architecture, allowing to overcome the limitations of “von Neumann bottleneck” to increase the efficiency of solving intellectual problems [8,9,10,11]. Neuromorphic systems are inspired by biology and are composed of many elements, the functionality of which is similar to some of the basic functions of the human brain. Computations and information storage are carried out throughout the neuromorphic system, and not in individual nodes of the system, as is the case with the von Neumann architecture [12]. At the same time, many parallel computing elements of neurons (about 10^11^) provide high performance in solving problems in real time [13]. The principle of the neuromorphic system also turns out to be different in comparison with traditional computing systems, programming is replaced by learning, i.e., the neuromorphic system learns to solve problems [14,15]. The learning process itself consists in adjusting the weighting coefficients of neurons, which ensures high noise immunity and fault tolerance in solving a number of problems related to pattern recognition, adaptive control, forecasting, and diagnostics, the solution of which takes an order of magnitude longer on traditional computing systems [16,17,18]. Moreover, the result of the neuromorphic system’s work is weakly dependent on the malfunction of an individual neuron. This makes them attractive for use in onboard intelligent systems.

The neuromorphic system can be partially implemented at the software level, but its hardware implementation would open significantly more possibilities in creating systems that imitate the work of the human brain [19,20,21]. To implement the operation of a neuron at the hardware level, a number of requirements are imposed on the element base in terms of energy efficiency, as well as the presence of non-volatility and multibit properties (the ability of an element to take three or more stable states). One of the ways to implement a neuromorphic system at the hardware level is to manufacture an array of memristors using cross-bar technology [22,23,24]. In this case, each memristor will act as a biological neuron, and the connecting contact will act as a synapse. Today there are several types of memristors, the main of which are ferroelectric nonvolatile memory FeRAM [25,26], magnetoresistive nonvolatile memory MRAM [27,28], memory with a change in the phase composition PRAM [29], and nonvolatile resistive memory ReRAM [30,31,32,33,34]. For the manufacture of neuromorphic systems, the latter is the most promising in terms of energy efficiency and multibit rate. The principle of operation of ReRAM is based on the resistive switching effect, i.e., a change in resistance between the states of high resistance (R_HRS_) and low resistance (R_LRS_) due to the formation and destruction of a nanoscale conduction channel in the bulk of the oxide film under an external electric field [35,36,37,38]. Nanoscale conduction channel consists of many oxygen vacancies, the generation or recombination of each of which leads to the emergence of a new resistive state, which makes it possible to create a neuromophilic system with a potentially high degree of multibitness. The effect of resistive switching is demonstrated by many metal oxides (TiO_x_, ZnO, NiO, HfO_x_, VO_x_), of which vanadium oxide is especially distinguished to create neuromorphic systems, primarily due to low switching values and high values of the HRS/LRS ratio [39,40,41,42].

However, there are no systematic studies of the influence of geometric and electrophysical parameters on the effect of resistive switching for the creation of neuromorphic systems based on vanadium oxide ReRAM elements. There are many methods for producing thin films, such as magnetron sputtering [43], atomic layer deposition [44], thermal evaporation [45], chemical vapor deposition [46], and pulsed laser deposition (PLD) [47,48]. Since PLD has a number of advantages over other methods of obtaining thin films of metal oxides [49,50,51] it was used to prepare thin films of vanadium oxide. Moreover, the PLD method is promising for the manufacture of prototyping elements of ReRAM neuromorphic systems, since it allows the formation of films of metal oxides in a wide range of parameters. In PLD, the composition and properties of the deposited layers are largely determined by the pressure in the growth chamber, the composition of the background pressure [52], and the deposition duration [53]. Thus, the purpose of this work is to study the influence of geometric and electrophysical parameters on the effect of resistive switching in vanadium oxide films obtained by pulsed laser deposition.

## 2. Materials and Methods

**Figure 1:**
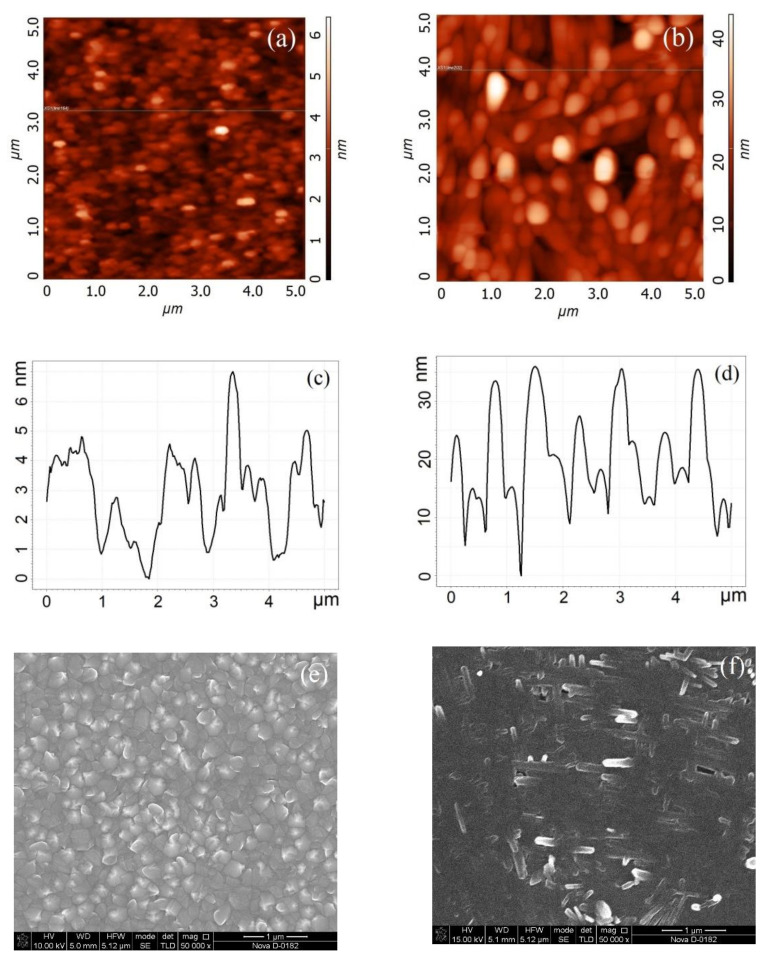
Vanadium oxide film surface formed at 10,000 pulse number (**a**,**c**,**e**) and at 60,000 pulse number (**b**,**d**,**f**): (**a**,**b**)—AFM-images; (**c**,**d**)—AFM cross-section; (**e**,**f**)—SEM-image.

Oxide vanadium thin films were fabricated using a Pioneer 180 pulsed laser deposition system (Neocera Co., Beltsville, MD, USA) equipped with a KrF excimer laser with a wavelength of 248 nm and an energy of 200 mJ (Figure 1). Si wafers with crystallographic orientation (100) were used as substrates. TiN 70 ± 14 nm thick bottom electrode was formed by PLD method under the following conditions: substrate temperature 700 °C, number of pulses 15,000, frequency 10 Hz, argon pressure 1 Torr.

**Figure 2:**
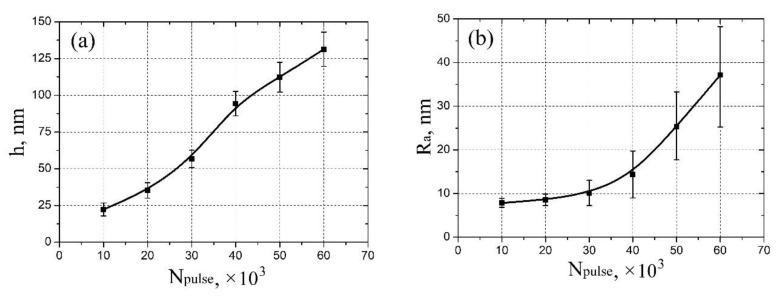
Experimental study of vanadium oxide film geometrical parameters dependence on pulse number: (**a**)—thickness; (**b**)—surface roughness.

**Figure 3:**
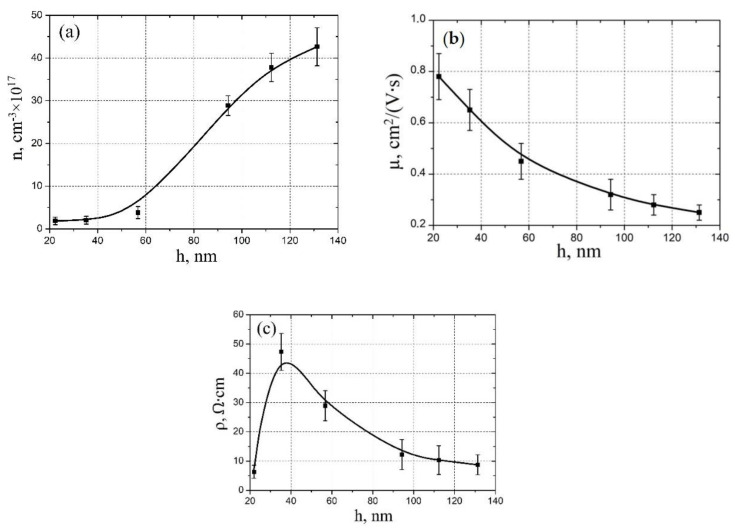
Experimental study electrophysical parameters dependence on vanadium oxide film thickness: (**a**)—electron concentration; (**b**)—electron mobility; (**c**)—resistivity.

To experimentally study the effect of the pulse number on the morphological and electrophysical parameters of vanadium oxide films, 6 samples were fabricated on TiN/Si structure under the following conditions: substrate temperature 800 °C, laser frequency 10 Hz, oxygen pressure 3 × 10^−4^ Torr. The samples were made with a different number of pulses in the range from 10,000 to 60,000 with a step of 10,000 pulses. Based on the obtained experimental results, the dependences of geometric (film thickness, surface roughness) parameters on the pulse number (Figure 2) and electrophysical (electron concentration, electron mobility, resistivity) on film thickness (Figure 3) were established.

**Figure 4:**
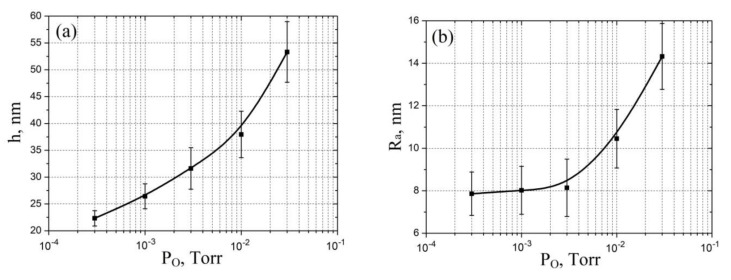
Experimental study of vanadium oxide film geometrical parameters dependence on oxygen pressure: (**a**)—thickness; (**b**)—surface roughness.

**Figure 5:**
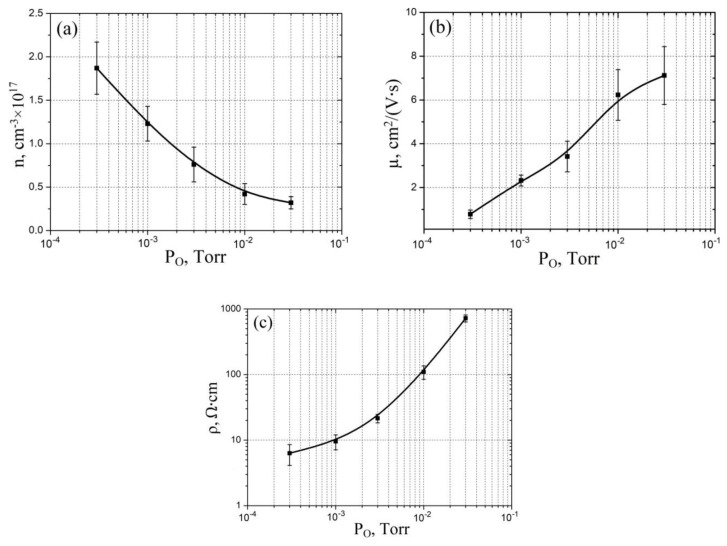
Experimental study of vanadium oxide film electrophysical parameters dependence on oxygen pressure: (**a**)—electron concentration; (**b**)—electron mobility; (**c**)—resistivity.

To experimentally study the effect of oxygen pressure on the morphological and electrophysical parameters of vanadium oxide films, five samples were prepared on TiN/Si structure under the following modes: substrate temperature 800 °C, laser frequency 10 Hz, number of pulses: 10,000. The samples were made at different oxygen pressures: 3 × 10^−4^ Torr, 1 × 10^−3^ Torr, 2 × 10^−3^ Torr, 1 × 10^−2^ Torr, and 3 × 10^−2^ Torr. Based on the obtained experimental results, the dependences of geometric (film thickness, surface roughness) (Figure 4) and electrophysical (electron concentration, electron mobility, resistivity) parameters on oxygen pressure (Figure 5) were established.

Geometric parameters of the vanadium oxide films were studied by atomic force microscopy (AFM) in the semicontact mode using the Ntegra Probe Nanolaboratory (NT-MDT, Zelenograd, Russia) and a commercial cantilever NSG11 with 255 kHz resonant frequency and 11.8 N/m spring constant. Vanadium oxide film thickness was determined using AFM by scanning of (vanadium oxide)/TiN interface. Processing of the results was carried out using the «Image Analysis 2.0» software package. Vanadium oxide film structure was investigated using Nova NanoLab 600 raster electron microscope (FEI Company, Hillsboro, OR, USA).

The electrophysical parameters of vanadium oxide films were studied using an Ecopia HMS-3000 Hall effect system (Ecopia Co., Anyang, Korea).

**Figure 6:**
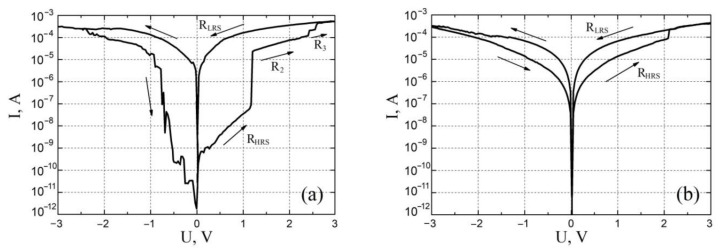
*I-V* characteristics of vanadium oxide films with different film thickness: (**a**)—22.3 ± 4.4 nm; (**b**)—131.7 ± 14.4 nm.

**Figure 7:**
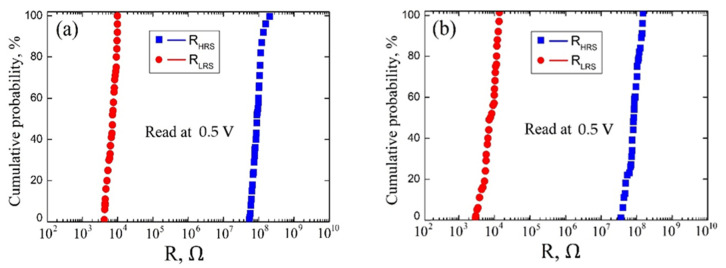
Cumulative probability data for the resistances of R_HRS_ and R_LRS_ for vanadium oxide films with 22.3 ± 4.4 nm film thickness: (**a**)—uniformity test; (**b**)—homogeneity test.

The resistive switching effect in vanadium oxide films was studied using a Keithley 4200-SCS semiconductor parameter analyzer (Keithley Instruments, Solon, OH, USA) and an EM-6070A submicron probe system (Planar, Republic of Belarus). TiN bottom electrode (BE) was grounded, W probe with diameter about 100 nm was used as the top electrode (TE). The compliance current was set to 0.7 mA to avoid thermal breakdown of vanadium oxide films. As a result, current-voltage (*I-V*) curves were obtained in the sweep voltage range from −3 V to 3 V for samples with a thickness of 22.3 ± 4.4 nm and 131.7 ± 14.4 nm at different points on the surface of the vanadium oxide film (Figure 6). Based on TE experimental results, the uniformity test (study of resistance switching at one point on the surface of a vanadium oxide film) and homogeneity test (study of resistance switching at different points on the surface of a vanadium oxide film) were carried out (Figure 7).

## 3. Results and Discussion

Figure 1 shows the results of experimental studies of the morphology of a vanadium oxide film with thicknesses 22.3 ± 4.4 nm and 131.7 ± 14.4 nm. It is shown that a film with a thickness of 22.3 ± 4.4 nm (10,000 pulse number) is granular (Figure 1e), when a film with a thickness of 131.7 ± 14.4 nm (60,000 pulse number) (Figure 2f) has a nanorod structure on the surface. This result can be explained by the dominance of different mechanisms of thin film growth with increasing vanadium oxide film thickness during the pulsed laser deposition process [54].

Based on the obtained experimental results, the dependences of the thickness and surface roughness of vanadium oxide films on the pulse number were established (Figure 2). It was shown that an increase in the pulse number from 10,000 to 60,000 leads to an increase in the thickness from 22.3 ± 4.4 nm to 131.7 ± 14.4 nm (Figure 2a), and to an increase in the surface roughness from 7.8 ± 1.1 nm to 37.1 ± 11.2 nm (Figure 2b). It should be noted that an increase in the pulse number may lead to a gradual transition of the vanadium oxide film from a granular structure to nanorod structure. One can assume that the possible mechanism for this phenomenon consists of two-stage process [54]. At the first stage, high-density crystalline nuclei are grown on the substrate surface, forming a granular film; at the second stage, due to the high surface energy of the substrate plane, the growth of the vanadium oxide film in one of the directions dominate, as a result nanorods are formed.

Figure 3 shows the results of experimental studies of thickness influence on the electrophysical properties of vanadium oxide films. It was shown that an increase in the thickness from 22.3 ± 4.4 nm to 131.7 ± 14.4 nm leads to an increase in the electron concentration of vanadium oxide from (1.87 ± 0.32) × 10^17^ cm^−3^ to (42.64 ± 4.46) × 10^17^ cm^−3^ (Figure 3a) and a decrease in the mobility of electrons from 0.78 ± 0.09 cm^2^/(V·s) to 0.25 ± 0.03 cm^2^/(V·s) (Figure 3b). This result can be explained by the fact that an increase in the pulse number and, therefore, in the thickness of the vanadium oxide film, leads to a decrease in the grain diameter [55]. This leads to an increase in grain density and the area of grain boundaries, which are defects with destroyed V-O bonds (oxygen vacancies) and has an additional number of electrons [56]. Therefore, we observe an increase in the electron concentration with an increase in the pulse number. A decrease in the mobility of electrons with an increase in the pulse number can also be explained by an increase in grain boundaries, which are an additional potential barrier for electrons. In addition, the analysis of the obtained results showed that an increase in the thickness of the vanadium oxide film from 22.3 ± 4.4 nm to 35.23 ± 5.26 nm leads to an increase in the resistivity from 6.32 ± 2.21 Ω·cm to 47.32 ± 6.32 Ω·cm. A further increase in the vanadium oxide film thickness from 35.23 ± 5.26 nm to 131.32 ± 11.64 nm leads to a decrease in the resistivity from 47.32 ± 6.32 Ω·cm to 8.74 ± 3.43 Ω·cm (Figure 3c).

This result can be explained also by the dominance of different growth mechanisms with different film thickness. The grain diameter has a complex dependence on the thickness, so with an increase in the thickness of the vanadium oxide film to about 40 nm, a decrease in the grain diameter of the granular film is observed, and a further increase in the thickness leads to an increase in the grain diameter with the formation of nanorods [54]. Energies of particles in the laser plume are different, and when hitting the substrate, these particles can interact with each other in different ways, with the formation of grains of different sizes. It is important to note that changes in the size of grains are associated not only with an increase or decrease in their area, but also with a change in their shape and location relative to each other. It can be assumed that the maximum value of the resistivity (Figure 3c) corresponds to the maximum area of grain boundaries, with different content of defects, charge carriers, and, therefore, resistance. It can be assumed that an increase in the resistivity from 6.32 ± 2.21 Ω·cm to 47.32 ± 6.32 Ω·cm with an increase in the film thickness from 22.32 ± 4.43 nm to 35.23 ± 5.26 nm is associated with a significant contribution of bulk conduction to the total conduction, along with grain-boundary conduction [57]. In this case, an increase in the volume fraction of a grain and an increase in the distance of overcoming the charge carriers between the grain boundaries can lead to an increase in the resistivity of the vanadium oxide film. A decrease in the resistivity from 47.32 ± 6.32 Ω·cm to 8.74 ± 3.43 Ω·cm with an increase in the film thickness from 35.23 ± 5.26 nm to 131.32 ± 11.64 nm can be associated with a change in the morphology of the film from granular structure to nanorod structure and dominance of rod boundary conduction over bulk conduction.

Analysis of the experimental results of studying the influence of oxygen pressure on geometric parameters showed that an increase in oxygen pressure from 3 × 10^−4^ Torr to 3 × 10^−2^ Torr leads to an increase in the film thickness from 22.32 ± 1.43 nm to 53.32 ± 5.65 nm (Figure 4a) and an increase in surface roughness from 7.8 ± 1.1 nm to 14.3 ± 1.5 nm (Figure 4b). This result can be explained by an increase in the number of oxygen atoms with increasing pressure, which leads to an increase in the formation of the number of V-O bonds with the formation of vanadium oxide atoms, and, finally, to an increase in the film thickness and surface roughness [54]. Detailed description of phase composition and stoichiometry of vanadium oxide obtained by pulsed laser deposited are presented in [58].

Figure 5 shows the results of experimental studies of oxygen pressure on the electrophysical properties of vanadium oxide films. It was shown that an increase in the oxygen pressure from 3 × 10^−4^ Torr to 3 × 10^−2^ Torr leads to a decrease in the electron concentration of vanadium oxide from (1.87 ± 0.32) × 10^17^ cm^−3^ to (0.32 ± 0.07) × 10^17^ cm^−3^ (Figure 5a), an increase in the electron mobility from 0.78 ± 0.09 cm^2^/(V·s) to 7.12 ± 1.32 cm^2^/(V·s) (Figure 5b), and an increase in the resistivity from 6.32 ± 2.21 Ω·cm to 723.74 ± 89.21 Ω·cm (Figure 5c). This result can be explained by the fact that the concentration of electrons in the vanadium oxide film is directly proportional to the concentration of vanadium atoms, which, in turn, is inversely proportional to the oxygen pressure. This assumption is confirmed by the value of the resistivity of the films obtained at low (less than 0.003 Torr) oxygen pressures, which are comparable to the resistivity of metals. In addition, at different oxygen pressures, the formation of oxide phases with different stoichiometric ratios of vanadium and oxygen is possible. The combination of these phases also affects the electrical properties of vanadium oxide films. Therefore, an increase in the oxygen pressure leads to an increase in phases with a high oxygen content (V_2_O_3_, V_2_O_5_), which, in turn, leads to a decrease in the electron mobility and an increase in the resistivity of the vanadium oxide film with an increase in the oxygen pressure.

The effect of resistive switching was studied on two films with different structures: granular and nanorod. For the granular structure films, a film with a thickness of 22.3 ± 4.4 nm was chosen, since it has the lowest roughness and resistivity, which is important for fabricating ReRAM neuromorphic structures with low energy consumption and a high degree of element integration. A film with a thickness of 131.7 ± 14.4 nm with the lowest resistivity was also selected from the nanorod structure films.

An analysis of the results obtained for studying the effect of resistive switching showed that a film with a thickness of 22.3 ± 4.4 nm exhibits a bipolar effect of resistive switching and has 4 regions: R_HRS_ at voltages from 0 V to 1.2 ± 0.1 V, R_2_ at voltages from 1.2 ± 0.1 V to 2.3 ± 0.1 V, R_3_ at voltages from 2.3 ± 0.1 V to 2.6 ± 0.1 V and R_LRS_ at voltages above 2.6 ± 0.1 V. The R_HRS_ was (89.42 ± 32.37) × 10^6^ Ω at 0.5 V read voltage, R_2_ was (32.53 ± 12.73) × 10^3^ Ω at 1.5 V read voltage, R_3_ was (8.47 ± 1.34) × 10^3^ Ω at 2.5 V read voltage V, R_LRS_ was equal to (6.34 ± 2.34) × 10^3^ Ω with a read voltage of 0.5 V. The ratio R_HRS_/R_LRS_ was about 14,104. This result can be associated with the phase inhomogeneity of the vanadium oxide film over the thickness. If we assume that in the process of PLD, with an increase in the pulse number, an increase in the number of oxygen atoms interacting with vanadium atoms occurs, this increase in the deposition time leads to the formation of oxides of higher order. As a result, the vanadium oxide film consists of several phases with different values of electrophysical parameters. In this case, the resistivity of the vanadium oxide film increases from the VO phase to the V_2_O_5_ phase (from the lower contact (BE) to the upper contact (TE)). This leads to the fact that the activation energy of oxygen atoms for the formation of a pair of O^−2^ (oxygen ions) and V_O_ (oxygen vacancy) also increases when moving from the film region with the VO phase to the film region with the V_2_O_5_ phase. Thus, to generate an oxygen vacancy in each phase, it is required to apply an external electric field above a certain value, which will impart energy to oxygen atoms above the activation energy. Based on this, it can be assumed that the R_HRS_ for the *I–V* characteristic in Figure 6a corresponds to the situation when oxygen vacancies are concentrated near the bottom contact in the VO phase. At a voltage of 1.2 ± 0.1 V, oxygen vacancies are formed in the VO_2_ phase and *IV* curve goes to the R_2_ region. At a voltage of 2.3 ± 0.1 V, the oxygen atoms receive sufficient energy to form a pair of O^−2^ and VO in the V_2_O_3_ phase, *IV* curve goes to the R_3_ region. At a voltage of 2.6 ± 0.1 V, the oxygen vacancies reach the upper contact and the film transforms into the R_LRS_ state. It can also be seen from the analysis of the *I–V* characteristics that at negative voltages, no plateaus are observed on the curve, as at positive ones. This may be due to the features of resistive switching from the R_HRS_ state to the R_LRS_, in particular, with the dominance of the temperature gradient over the electric field gradient and the concentration gradient of oxygen vacancies, which leads to the excessive release of Joule heat in the film volume and destruction of the entire nanoscale conduction channel, rather than its separate section [58].

In addition, an analysis of the obtained results of studying the resistive switching showed that a film with a thickness of 131.7 ± 14.4 nm exhibits a bipolar effect of resistive switching and has 2 resistive states: at a voltage of 2.3 ± 0.2 V, the film switched from the R_HRS_ state to R_LRS_, and at a voltage of −2.8 ± 0.1 the film was switched back to the R_HRS_ state (Figure 6b). The resistances of the R_HRS_ and R_LRS_ were (50.72 ± 13.42) × 10^3^ Ω and (12.34 ± 3.75) × 10^3^ Ω at a reading voltage of 1.0 V, respectively. The ratio R_HRS_/R_LRS_ was about 4. Analysis of the literature data showed that the main mechanism of conduction in oxide nanorods is the correlated barrier hopping model, according to which current transfer occurs due to the hopping of charge carriers between defects over a potential barrier separating them [59,60,61].

An analysis of the experimental results of studying the uniformity of resistive switching in a vanadium oxide film with a thickness of 22.3 ± 4.4 nm showed that R_LRS_ varied in the range from 4.27 × 10^3^ Ω to 9.88 × 10^3^ Ω, and R_HRS_ varied in the range from 5.32 × 10^7^ Ω to 1.23 × 10^8^ Ω. The mean values R_LRS_ = (6.34 ± 2.34) × 10^3^ Ω and R_HRS_ = (89.42 ± 32.37) × 10^6^ Ω corresponded to the probabilities of 41% and 52%, respectively (Figure 7a). An analysis of the experimental results of studying the homogeneity of resistive switching in a vanadium oxide film with a thickness of 131.7 ± 14.4 nm showed that R_LRS_ varied in the range from 2.93 × 10^3^ Ω to 1.37 × 10^4^ Ω, and R_HRS_ varied in the range from 3.64 × 10^7^ Ω to 1.51 × 10^8^ Ω. The mean values R_LRS_ = (6.34 ± 2.34) × 10^3^ Ω and R_HRS_ = (89.42 ± 32.37) × 10^6^ Ω corresponded to the probabilities of 37% and 61%, respectively (Figure 7b).

The different range of R_HRS_ and R_LRS_ values for uniformity and homogeneity can be explained by different values of the vanadium oxide film thickness, as well as different concentrations and distribution profiles of oxygen vacancies in the volume at different points on the vanadium oxide surface.

## 4. Conclusions

The paper presents the results of experimental studies of the influence of the PLD control parameters on the morphological and electrophysical parameters of vanadium oxide films. It is shown that an increase in the pulse number from 10,000 to 60,000 leads to an increase in the thickness of the vanadium oxide film from 22.3 ± 4.4 nm to 131.7 ± 14.4 nm, surface roughness from 7.8 ± 1.1 nm to 37.1 ± 11.2 nm, and electron concentration from (1.87 ± 0.32) × 10^17^ cm^−3^ to (42.64 ± 4.46) × 10^17^ cm^−3^, and a decrease in the electron mobility from 0.78 ± 0.09 cm^2^/(V·s) to 0.25 ± 0.03 cm^2^/(V·s). It is shown that an increase in the film thickness from 22.3 ± 4.4 nm to 35.23 ± 5.26 nm leads to an increase in the resistivity from 6.32 ± 2.21 Ω·cm to 47.32 ± 6.32 Ω·cm, and an increase in the film thickness from 35.23 ± 5.26 nm to 131.32 ± 11.64 nm leads to a decrease in the specific resistance from 47.32 ± 6.32 Ω·cm to 8.74 ± 3.43 Ω·cm. This result can be explained by the predominance of different growth mechanisms on samples with different film thicknesses of vanadium oxide, which leads to the formation of films with different structures.

An analysis of the obtained experimental results showed that an increase in the oxygen pressure from 3 × 10^−4^ Torr to 3 × 10^−2^ Torr leads to an increase in the thickness of the vanadium oxide film from 22.32 ± 1.43 nm to 53.32 ± 5.65 nm, and the surface roughness from 7.8 ± 1.1 nm to 14.3 ± 1.5 nm, electron mobility from 0.78 ± 0.09 cm^2^/(V·s) to 7.12 ± 1.32 cm^2^/(V·s), resistivity from 6.32 ± 2.21 Ω·cm to 723.74 ± 89.21 Ω·cm, and a decrease in concentration electrons from (1.87 ± 0.32) × 10^17^ cm^−3^ to (0.32 ± 0.07) × 10^17^ cm^−3^. This result can be explained by the formation of oxide phases with different stoichiometric ratios of vanadium and oxygen at different oxygen pressures.

An analysis of the obtained experimental results of studying the effect of resistive switching showed that a vanadium oxide film 22.3 ± 4.4 nm thick exhibits a bipolar effect of resistive switching and has 4 different resistive states. The R_HRS_ was (89.42 ± 32.37) × 10^6^ Ω at 0.5 V read voltage, R_2_ was (32.53 ± 12.73) × 10^3^ Ω at 1.5 V read voltage, R_3_ was (8.47 ± 1.34) × 10^3^ Ω at 2.5 V read voltage V, R_LRS_ was equal to (6.34 ± 2.34) × 10^3^ Ω with a read voltage of 0.5 V. The ratio R_HRS_/R_LRS_ was about 14,104. This result can also be associated with the phase inhomogeneity of the vanadium oxide film over the thickness. As a result, in different parts of the oxide film volume, the activation energy of oxygen atoms for the formation of a pair O^−2^ and V_O_ has different values. Therefore, imparting additional energy to atoms by increasing the sweep voltage amplitude leads to a sharp generation of oxygen vacancies in a particular phase and a sharp change in the resistance of the oxide film. At a voltage of 1.2 ± 0.1 V, oxygen vacancies are formed in the VO_2_ phase and the vanadium oxide film transforms into the R_2_ state. At a voltage of 2.3 ± 0.1 V, the oxygen atoms receive sufficient energy to form a pair of O^−2^ and VO in the V_2_O_3_ phase, and the vanadium oxide film transforms into the R_3_ state. At a voltage of 2.6 ± 0.1 V, the oxygen vacancies reach the upper contact and the film transforms into the R_LRS_ state.

Moreover, an analysis of the obtained results of studying the resistive switching showed that a film with a thickness of 131.7 ± 14.4 nm exhibits a bipolar effect of resistive switching and has two resistive states: R_HRS_ = (50.72 ± 13.42) × 10^3^ Ω and R_LRS_ = (12.34 ± 3.75) × 10^3^ Ω at a reading voltage of 1.0 V. The probable mechanism of resistive switching in the film with a nanorod structure can be described by the correlated barrier hopping model.

An analysis of the experimental results of studying the resistive switching in a vanadium oxide film with a thickness of 22.3 ± 4.4 nm showed that R_LRS_ varied in the range from 4.27 × 10^3^ Ω to 9.88 × 10^3^ Ω, and R_HRS_ varied in the range from 5.32 × 10^7^ Ω to 1.23 × 10^8^ Ω for uniformity test; R_LRS_ varied in the range from 2.93 × 10^3^ Ω to 1.37 × 10^4^ Ω, and R_HRS_ varied in the range from 3.64 × 10^7^ Ω to 1.51 × 10^8^ Ω for homogeneity test.

The results can be used in the manufacture of new-generation micro- and nanoelectronics elements to create ReRAM elements of neuromorphic systems based on vanadium oxide thin films.

## Data Availability

Not applicable.
